# Defining tuberculosis vulnerability based on an adapted social determinants of health framework: a narrative review

**DOI:** 10.1080/17441692.2023.2221729

**Published:** 2023-06-11

**Authors:** Shishi Wu, Stefan Litvinjenko, Olivia Magwood, Xiaolin Wei

**Affiliations:** a Dalla Lana School of Public Health, University of Toronto, Toronto, Canada; b Global Implementation Science Lab, University of Toronto, Toronto, Canada; c Bruyère Research Institute, Ottawa, Canada; d Interdisciplinary School of Health Sciences, Faculty of Health Sciences, University of Ottawa, Ottawa, Canada

**Keywords:** Tuberculosis, vulnerability, vulnerable populations, social determinants of health

## Abstract

The World Health Organization’s new End TB Strategy emphasises socioeconomic interventions to reduce access barriers to TB care and address the social determinants of TB. To facilitate developing interventions that align with this strategy, we examined how TB vulnerability and vulnerable populations were defined in literature, with the aim to propose a definition and operational criteria for TB vulnerable populations through social determinants of health and equity perspectives. We searched for documents providing explicit definition of TB vulnerability or list of TB vulnerable populations. Guided by the Commission on the Social Determinants of Health framework, we synthesised the definitions, compiled vulnerable populations, developed a conceptual framework of TB vulnerability, and derived definition and criteria for TB vulnerable populations. We defined TB vulnerable populations as those whose context leads to disadvantaged socioeconomic positions that expose them to systematically higher risks of TB, but having limited access to TB care, thus leading to TB infection or progression to TB disease. We propose that TB vulnerable populations can be determined in three dimensions: disadvantaged socioeconomic position, higher risks of TB infection or progression to disease, and poor access to TB care. Examining TB vulnerability facilitates identification and support of vulnerable populations.

## Abbreviation

CSDH: Commission on Social Determinants of Health
ECDC: The European Centre for Disease Prevention and Control
IDP: Internally displaced person
MSM: Men who have sex with men
TB: Tuberculosis
WHO: World Health Organization

## Introduction

Tuberculosis (TB) has been long regarded as a social disease. Even before the discovery of the tubercle bacillus by Robert Koch in 1882, a correlation between TB incidence and poverty was observed, as the majority of people who developed or died from TB were among the working class and poor families (Paluzzi, 2004). In the 1950s, Dubos, a microbiologist by training, commented that TB ‘is a social disease … its understanding demands that the impact of social and economic factors on the individual be considered as much as the mechanisms by which tubercle bacilli cause damage to the human body’ (Dubos & Dubos, 1952). Subsequently, a large body of epidemiological evidence has quantified the association between TB incidence and socio-economic determinants (Hargreaves et al., 2011; Rocha et al., 2011; Uplekar et al., 2015; Wingfield et al., 2014). For example, poor ventilation, overcrowded living conditions, poverty and malnutrition all increase the risk of exposure to TB or progression to TB disease (Baker et al., 2008; Boccia et al., 2009; Hill et al., 2006) (Cegielski & McMurray, 2004; Jaganath & Mupere, 2012; Oxlade & Murray, 2012; Santos et al., 2007). Many additional studies have supported the notion that the TB burden follows a strong socio-economic gradient, both within and across countries, with the poor having the highest risk of developing TB (Holtgrave & Crosby, 2004; Lopez De Fede et al., 2008; Muniyandi et al., 2007).

To guide the global response to ending TB as a public health problem (Rocha et al., 2011; Uplekar et al., 2015; Wingfield et al., 2016), in 2015, the World Health Organization (WHO) published the End TB Strategy, in which achieving universal access to TB care, addressing weaknesses in health systems and the social determinants of TB are key features (Uplekar et al., 2015). The strategy explicitly includes reference to health and social sector policies to strengthen national responses to TB as a key pillar, highlighting the importance of implementing socioeconomic interventions to reduce access barriers to TB care, strengthen social protection, and address the social determinants of TB (Uplekar et al., 2015). However, terms such as high-risk populations, marginalised populations, and vulnerable populations are sometimes used interchangeably to indicate target groups for such interventions. Therefore, developing a uniform definition of vulnerability within the TB context is crucial in identifying the communities and populations that are at a higher risk of contracting TB, and differentiating it from related concepts. This is an essential step in addressing the root causes of differential TB exposure and guaranteeing that individuals with the greatest need can access top-notch TB healthcare.

Various definitions of vulnerability already exist. For example, vulnerability has been defined by ten Have as ‘the state of susceptibility to harm from exposure to stresses associated with environmental and social change and from the absence of capacity to adapt’ (ten Have, 2018), whereas Rogers et al., state that vulnerability arises from biological, social, political, environmental, and cultural sources (Rogers et al., 2012). Allotey et al., have proposed that vulnerability is caused by individuals’ inability to protect themselves from harm and that vulnerability experienced by groups is shaped by shared ethnic, cultural, and social similarities (Allotey et al., 2012). According to Wisner, vulnerability is created by socioeconomic and political processes that expose individuals to different levels of harms (Wisner et al., 2014). Despite some differences in these definitions, vulnerability is often examined through equity, human rights, and bioethics lenses, and is rooted in inequalities in power, knowledge, and resources within societies, potentially resulting in harms.

Our narrative review aimed to examine how TB vulnerability and vulnerable populations are defined and characterised in the literature. Based on this review, we proposed a definition and criteria for TB vulnerable populations. We also compiled a list of TB vulnerable populations identified from the literature and aligned their attributes to the Commission on the Social Determinants of Health (CSDH) framework to develop a conceptual framework of TB vulnerability (World Health Organization, 2010).

## Methods

We employed the narrative review method following the steps outlined by Ferrari, instead of a scoping or systematic review, because it is useful and practical when tracking the development of scientific principles or concepts (Ferrari, 2015).

### Literature search

We developed systematic search strategies with a medical librarian to identify documents published in English, French, or Chinese that included definitions or criteria of TB related vulnerability. We searched the following databases, for the period 1 January 2010–31 October 2021: Medline, EMBASE, and the Cochrane Database for Systematic Reviews. We used a combination of keywords and subject headings to combine concepts of TB, and vulnerability. Complete search strategies for each database are available in Appendix 1. In addition, relevant journals, policy briefs, and technical proceedings (e.g. WHO and Stop TB Partnership guidelines and documents) were hand-searched. Reference lists of documents meeting the eligibility criteria were inspected for additional relevant information.

### Selection

The results of the literature search were imported into Covidence to facilitate selection of articles and documents to be included in the review using a two-phased approach. The inclusion and exclusion criteria are summarised in Table 1. An explicit definition of TB vulnerability or criteria to define vulnerability and/or a list of TB vulnerable populations, rather than simply a mention, were required for inclusion. We included documents published since the year 2010. This cut-off was selected to be sensitive to contemporary understandings of vulnerability. We followed a two-phase selection process using Covidence software. In phase one, capacity across four independent reviewers permitted title and abstract screening of all identified citations against our eligibility criteria (no article received more than two votes). We ensured all members were applying the eligibility criteria consistently by reviewing the first 100 citations individually, with conflicts for citations receiving ‘maybe-yes’ and ‘maybe-no’ votes resolved through discussion with the lead author (SL) In phase two, all full texts were assessed by one reviewer (SL.) and a sample of citations were assessed by a second reviewer (SW).

**Table 1.** Inclusion and exclusion criteria.

Inclusion criteria	Include documents in which an explicit definition of TB vulnerability or criteria to define vulnerability and/or a list of TB vulnerable populations were presented Include published documents, such as peer-reviewed journal articles, policy documents, reports, etc. Include documents published since the year 2010 Include documents in English, Chinese, and French
Exclusion criteria	Exclude documents which simply mention TB vulnerability and/or TB vulnerable populations without giving explicit definition and/or criteria and/or list of TB vulnerable populations Exclude documents which defined vulnerability relating to patients with multidrug-resistant tuberculosis or latent TB infection

### Synthesis

First, we synthesised the definitions of TB vulnerability in a narrative manner. We then compiled a list of vulnerable populations identified from the documents and aligned the attributes of these vulnerable populations to the social determinants of health that are indicated as constructs and sub-constructs in the CSDH framework (World Health Organization, 2010). Based on the synthesis of TB vulnerable populations identified from the literature, we developed a conceptual framework of TB vulnerability. We derived a definition of and operational criteria for determining TB vulnerable populations from the proposed conceptual framework accordingly.

The CSDH framework is underpinned by the ethical principles of health equity (World Health Organization, 2010). It posits that ‘social, economic, and political mechanisms give rise to a set of socioeconomic positions; these socioeconomic positions in turn shape specific determinants of health status reflective of people’s place within social hierarchies; based on their respective social status, individuals experience differences in exposure and vulnerability to health-compromising conditions’ (World Health Organization, 2010). The framework explains and illustrates how the broader structural determinants dictate the distribution of intermediary determinants of health, thus resulting in systematic differences in disease risks experienced across populations (World Health Organization, 2010).

The CDSH framework has five major constructs: context, socioeconomic positions, intermediary determinants, health system, health equity and outcomes. Context refers to a broad set of structural, cultural, and functional factors and mechanisms within a social system that create hierarchies and define individuals’ socioeconomic positions (World Health Organization, 2010). The context facilitates unequal distribution of power, resources, and opportunities. This process of creating inequality within societies is often portrayed as social stratification. Individuals’ positions in stratified societies can generally be referred to as their socioeconomic positions (World Health Organization, 2010). Common proxy indicators for socioeconomic position include income, education, occupation, and ethnicity (World Health Organization, 2010). These underlying structural determinants operate through a set of intermediary determinants of health, including material circumstances, psychosocial circumstances, behavioural and biological factors, which leads to differences in exposure to disease risks and accessibility to health systems, and ultimately shapes health outcomes (World Health Organization, 2010).

To align with the scope of this review, we adapted the CSDH framework to specifically focus on how socioeconomic position and intermediary determinants shape TB vulnerability in populations. This helped us understand the structural and intermediatory determinants that create inequalities and differential exposure to TB. With this approach, we identified TB vulnerable populations and developed equity-focused health policies and interventions.

## Results

### Definitions of TB vulnerability identified from the literature

We reviewed the titles and abstracts of 10,648 unique records. We found four documents that provided explicit definitions of vulnerability specifically in the TB context. These definitions highlight the impact of the broader socioeconomic determinants on TB vulnerability and are primarily grounded in human rights and equity. The WHO consolidated guidelines on TB screening state that TB disproportionately affects individuals who are already disadvantaged due to disease, their socioeconomic situation, or legal status, among other disadvantages, and these individuals are regarded as being vulnerable to TB (World Health Organization, 2021). In 2016, the Global Fund to Fight AIDS, TB and Malaria (Global Fund) defined vulnerable populations as ‘people whose situations or contexts make them especially vulnerable, or who experience inequality, prejudice, marginalisation, and limits on their social, economic, cultural and other rights’ (Greenall et al., 2017). Go et al., defined vulnerable populations as those having limited access to healthcare or lacking the economic resources to support costs associated with TB treatment (Go et al., 2018). The European Centre for Disease Prevention and Control (ECDC) characterises vulnerable populations as ‘those whose socioeconomic conditions or lifestyle makes it difficult to recognise TB symptoms, access health services, self-administer treatment and attend regular healthcare appointments’ (European Centre for Disease Prevention Control, 2016). It further acknowledged an increased risk of TB in these groups arising from ‘multiple socio-behavioural determinants that act at different levels and commonly exacerbate one another’ (European Centre for Disease Prevention Control, 2016).

### Proposed definition and criteria of TB vulnerable populations

We defined TB vulnerable populations as the following:

People whose context leads to disadvantaged socioeconomic positions that put them at systematically higher risk for TB, with limited access to appropriate or high quality TB care, thus with a higher likelihood of experiencing health inequalities, developing TB infection or progression to TB disease. While vulnerable populations may include persons who are overrepresented in measures of TB risk and/or burden, vulnerability fundamentally precedes risk, leading to an increased risk of exposure or progression to disease, or of poor outcomes, or all of these.

**Figure 1.**
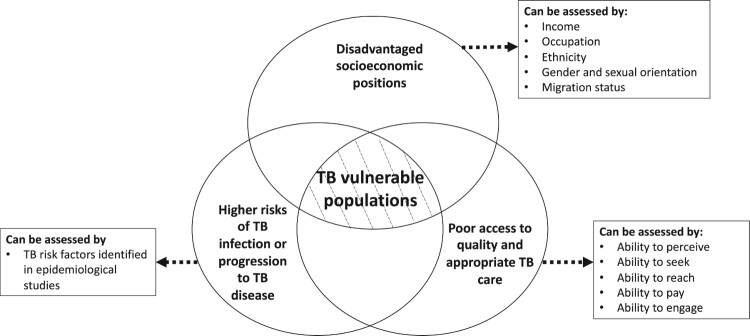
Criteria for determining populations vulnerable to TB.

In line with this definition, we propose the following *three dimensions* as criteria for identifying vulnerable populations (Figure 1):

1. **Disadvantaged socioeconomic positions**. Socio-economic position refers to individuals’ place within social hierarchies, and can be assessed by proxy indicators, such as income, occupation, ethnicity, gender or sexual orientation, and migration status. Disadvantaged socioeconomic positions can be reflected by lower income, having occupations with higher health risks but lower social standing, being in ethnic or sexual minorities, or from certain migrant groups.
2. **Higher risks of TB infection or progression to TB disease** are determined by a combination of intrinsic factors (such as the infectiousness of the index case) and external factors that increases exposure to TB or accentuates the progression from infection to disease (Narasimhan et al., 2013). TB risk factors can be identified in epidemiological studies which quantify the probability of TB infection in relation to TB exposure and the subsequent progression to TB disease.
3. **Poor access to quality or appropriate TB care.** Access refers to ‘the timely use of personal health services to achieve the best health outcomes’ (Millman, 1993), and can be assessed by five abilities – abilities to perceive, to seek, to reach, to pay, and to engage – which collectively represent individuals’ ability to interact with the health systems when seeking and receiving health services (Haldane et al., 2021). Ability to perceive indicates the participants’ knowledge and awareness of needing TB care. Ability to seek refers to knowledge of available healthcare options. Ability to reach refers to factors that may enable or deter individuals from physically reaching the care that they would like to access. Ability to pay refers to individuals’ capacity to pay for travel to health facilities and health services. Ability to engage indicates individuals’ interaction with healthcare providers and their access to health-related information (Haldane et al., 2021).

### List of TB vulnerable populations identified in the literature

We found 12 documents providing list of TB vulnerable populations, including: miners, sex workers, indigenous (First Nations) peoples, men who have sex with men, transgender individuals, refugees and internally displaced people, asylum seekers, migrant workers, the homeless, people residing in urban slums or informal settlements, incarcerated populations, nomadic population, people with HIV, people who use drugs and people with alcohol use disorders. The list of TB vulnerable populations aligned to the constructs and sub-constructs of the adapted CSDH framework is shown in Table 2.

**Table 2.** List of tuberculosis vulnerable populations aligned to the construct and sub construct of the social determinants of health framework.

Construct	Sub-construct	Vulnerable populations
Socioeconomic position	Occupation	Miners; sex workers
	Ethnicity	Indigenous populations
	Gender and sexual orientation	Men who have sex with men (MSM) and transgender individuals
	Migration	IDPs; asylum seekers; migrant workers;
Intermediary determinants	Material circumstances	People experiencing homelessness; people residing in urban slums or informal settlements; incarcerated populations; nomadic populations
	Behavioural and biological factors	People who use drugs; people with alcohol use disorders; people living with HIV

*Socioeconomic position* is a key construct in the CSDH, which can be determined by subconstructs, such as income, occupation, ethnicity, gender and sexual orientation, and migration status. Income is a direct indicator that measures material resources, which in turn influence material conditions that impact upon health. The WHO End TB Strategy, as well as other documents indicated that low-income is one of the sources contributing to vulnerability and a characteristic shared by many groups vulnerable to TB (Lönnroth et al., 2015; Nadjane Batista Lacerda et al., 2014; World Health Organization, 2001). Occupation not only determines individuals’ exposure to specific occupational risks, but also reflects individuals’ position in society related to their social standing, income, and education (Galobardes et al., 2006). In this review, groups defined as vulnerable populations based on their occupations included miners and sex workers (Heuvelings et al., 2018; Stop TB Partnership, 2017), as these occupations often have specific health risks but also are in disadvantaged socioeconomic positions that may be characterised by low income and education, marginalisation, and limited access to health care. Few documents grouped healthcare workers into key populations for TB control. However, in this review that examined vulnerability from a social determinants of health perspective, groups that are characterised by higher TB risks alone, such as healthcare workers, were considered high-risk groups from an epidemiological perspective, but not vulnerable populations. In the context of TB, indigenous (First Nations) populations were identified to be particularly vulnerable due to poverty, inadequate healthcare infrastructure, and cultural and social factors (Nadjane Batista Lacerda et al., 2014; The Global Fund, 2021). Indigenous populations often have lower socioeconomic status, which can lead to overcrowding, malnutrition, and limited access to healthcare, contributing to increased risks of TB infections. Additionally, stigma and discrimination against indigenous populations may prevent them from seeking medical care or following through with treatment. Gender and sexual orientation also often form the basis of discrimination, and sexual minorities often experience higher levels of stigma and stress (Meyer, 2003). These factors further enhance social disadvantages and, consequently, may increase the risks of negative health outcomes. In the reviewed documents, men who have sex with men (MSM) and transgender individuals were listed among TB vulnerable populations (Stop TB Partnership, 2017). Although migration is not a construct in the original CSDH framework, many recent studies argue that certain migrant groups are adversely affected by cultural and social isolation, which further complicates the effects of other socioeconomic positioning indicators (e.g. income, ethnicity, and education), ultimately impacting on health outcomes (Sardadvar, 2015). Our reviewed documents identified migrant workers, refugees, internally displaced persons (IDPs) and asylum seekers as vulnerable populations for TB (Dara et al., 2016; European Centre for Disease Prevention Control, 2016; Heuvelings et al., 2018; Lönnroth et al., 2015; Sulis et al., 2014; The Global Fund, 2021; World Health Organization, 2001, 2021).

*Intermediary determinants.* The underlying inequities resulting from differences in socioeconomic positions operate through a set of intermediary determinants of health, which are linked to individual-level physical conditions, health-related behaviours and physiological factors that shape health outcomes. In the CSDH framework, the main categories of intermediary determinants are material circumstances, behavioural and biological factors. Material circumstances include consumption potential (e.g. having the means to access and purchase food, clothes, and other consumer goods), physical working conditions, and the environment. These circumstances provide both resources for health and risks for health (World Health Organization, 2010). In the review, we found material circumstances, specifically the physical environment and living conditions, as a main factor for TB vulnerability. The reviewed documents defined homeless people (European Centre for Disease Prevention Control, 2016; Gupta et al., 2018; Heuvelings et al., 2018; Lönnroth et al., 2015; Sulis et al., 2014), people residing in urban slums (Shewade et al., 2019), prisoners (European Centre for Disease Prevention Control, 2016; Gupta et al., 2018; Heuvelings et al., 2018; Lönnroth et al., 2015; Sulis et al., 2014; World Health Organization, 2001, 2021), and nomadic populations (Shewade et al., 2019) to be vulnerable to TB. Behavioural factors can protect health (e.g. physical activity) or adversely affect health outcomes (e.g. smoking and drug use). Our reviewed documents identified individuals with harmful alcohol use (European Centre for Disease Prevention Control, 2016; Lönnroth et al., 2015; Sulis et al., 2014) and drug users as TB vulnerable populations (European Centre for Disease Prevention Control, 2016; Gupta et al., 2018; Heuvelings et al., 2018; Lönnroth et al., 2015; World Health Organization, 2001). Biological factors include genetic and epidemiological risk factors for the disease, which may also compound with social stigma and discrimination to prevent individuals from accessing appropriate TB care. We found that people living with HIV were included as TB vulnerable populations in the reviewed documents (Heuvelings et al., 2018; Lönnroth et al., 2015; Sulis et al., 2014; World Health Organization, 2001).

### Proposed conceptual framework of TB vulnerability

**Figure 2.**
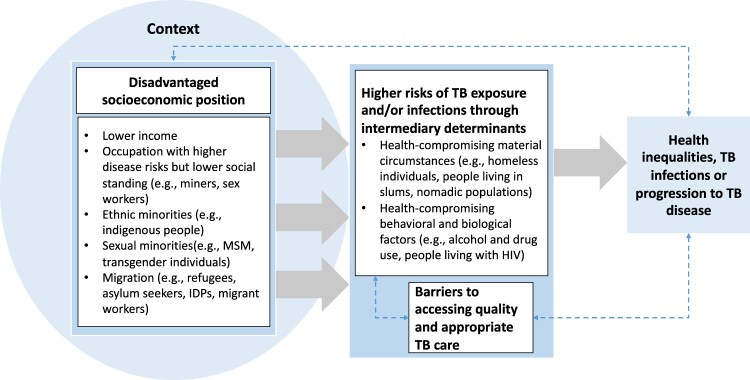
A conceptual framework of TB vulnerability based on adapted Commission on Social Determinants of Health (CSDH) framework.

Based on our synthesis of TB vulnerable populations through the adapted CSDH framework, we propose a conceptual framework of TB vulnerability in Figure 2. We suggest that TB vulnerability stems from social stratification that places individuals and groups into different socioeconomic positions. Populations in disadvantaged socioeconomic positions, indicated by lower income, high-risk occupations with low social status, ethnic or sexual minority status, or belonging to certain migrant groups, are more likely to be exposed to health-compromising material conditions and behaviours that increase their risk of TB exposure, progression, and limited access to quality TB care (Figure 2). These populations are also at higher risk of experiencing health inequalities and developing TB infection or disease.

## Discussion

In this review, we examined the existing definitions of TB vulnerability, provided a definition of TB vulnerability, and proposed that it can be determined by a composite of three dimensions: socioeconomic positions, risk of TB infection or disease, and access to appropriate TB care. We compiled a list of TB vulnerable populations identified in the literature, guided by the adapted CSDH framework. Based on this synthesis, we developed a conceptual framework of TB vulnerability that highlights that disadvantaged socioeconomic positions often lead to higher risks of TB exposure or of developing TB disease and poorer healthcare access among certain populations.

Similar to previous definitions of TB vulnerability that are grounded in health equity and provided by key global stakeholders working on TB (European Centre for Disease Prevention Control, 2016; Go et al., 2018; Greenall et al., 2017; World Health Organization, 2021), our proposed definition and conceptual framework of TB vulnerability reaffirms that the social determinants of health is an underlying contributor to TB vulnerability in populations, thus indicating the need to address these underlying social and economic factors that contribute to differential exposure to TB risks, affect access to TB care and also outcomes related to the disease. Our proposed definition and conceptual framework of TB vulnerability align with the principles highlighted in the End TB Strategy and strategic directions taken by key global stakeholders working on TB (Lönnroth et al., 2015). By adopting human rights and equity as principles for TB responses, the End TB Strategy calls for adaptation of services to the special needs of the most vulnerable groups and the development of multisectoral actions to address the structural determinants of health (Lönnroth et al., 2015). The Global Fund expanded the list of key and vulnerable populations in their 2017–2022 strategy, which states that strengthened efforts are needed to address the challenges posed by poor access to healthcare experienced by these populations (Greenall et al., 2017).

Identifying TB vulnerable populations is crucial for developing targeted interventions and policies that address their specific needs and underlying vulnerabilities, which can help mitigate the TB risks caused by socioeconomic inequalities (Lönnroth et al., 2015). To aid in this process, we have proposed operational criteria for identifying vulnerable populations based on our conceptual framework of TB vulnerability. Our criteria incorporate the dimension of ‘socioeconomic positions’ to reflect the equity perspective and broader structural considerations of TB vulnerability beyond epidemiological risk or burden alone. This differentiation from ‘high-risk groups’ or ‘key populations’ emphasises the need for a more nuanced approach. Moreover, we have also included ‘risk of TB infection or disease’ and ‘access to TB care’ dimensions, which acknowledge the contextual specificity of vulnerable populations and the local epidemiology of TB and healthcare systems (European Centre for Disease Prevention Control, 2016).

However, we acknowledge that TB vulnerability is complex and can be examined from different angles. It should be noted that our review and the development of the definition and conceptual framework of TB vulnerability were performed from a social determinants of health perspective and were based on the contemporary and common understanding of vulnerability in the available literature, with the underlying assumption that TB risk follows a socio-economic gradient (Holtgrave & Crosby, 2004; Lopez De Fede et al., 2008; Ten Have, 2014). Our proposed operational criteria for identifying populations vulnerable to TB are intended to be used by policymakers, program implementers, and researchers to select groups to be included in interventions or research studies that align with the principles of the End TB Strategy, and aim to address underlying inequalities contributing to increased TB exposures and risks, limit access to quality TB health services, and differential outcomes related to the disease. The proposed definition and criteria of TB vulnerable populations highlight the importance of considering the broader socioeconomic determinants of health that contribute to TB vulnerability. Therefore, policymakers and researchers in the field should bear in mind that vulnerability to TB is a complex and multifaceted concept that goes beyond individual risk factors. The principles of human rights and equity should be taken into account when developing and implementing policies, programs, and interventions to address TB vulnerability.

### Strengths and limitations

Our study has the following strengths. First, we provided a comprehensive overview of the current understanding of TB vulnerability, and its findings could inform the development of strategies to reduce TB burden and health inequalities. Second, the proposed operational definition and criteria for identifying TB vulnerable populations, may be helpful for future researcher, program implementers, and policymakers to develop human rights and equity centred TB interventions and programs. Finally, we used the CSDH framework as the theoretical underpinning for developing the definition and conceptualising of TB vulnerability, which provides a comprehensive and evidence-based approach to understanding the social determinants of health and how they contribute to health inequalities in the TB context.

Since the aim of this review was to provide an overview of a broad concept, we performed a narrative review, instead of a systematic or scoping review, thus our study is susceptible to the intrinsic limitations of narrative reviews. First, although we were missing dedicated search terms accounting for age, our search strategy was developed to be as sensitive as possible, as it consists of search terms for commonly known factors contributing to higher TB risks and a wide range of TB high-risk populations literature. Interestingly, there was limited literature to support persons with mental illness as a vulnerable population for TB, and we did not find documents specifically listed adolescents and the elderly as TB vulnerable populations. Conversely, we retrieved results on ‘high-risk populations’ which ultimately did not meet our criteria for TB vulnerability, such as people with diabetes, pregnant women, and healthcare workers. As screening for both definitions of TB vulnerability and related populations was done based on the same literature in tandem, it was challenging to accurately account for, and delineate nearly 20 population candidates a priori. Second, because many included documents were reports or guidelines, we were unable to assess the data quality using quality appraisal tools that are often used for assessing peer-reviewed studies as part of reviews. Third, our synthesis of data was not conducted based on a systematic data extraction and synthesis protocol; instead, we performed a narrative synthesis of information relevant to the aim of the study from documents selected by reviewers. Fourth, we limit our searches to low and middle-income countries, because the majority of the global burden of tuberculosis occurs in these countries. Finally, our review did not include national TB strategic plans, in which country-specific TB vulnerable populations may be presented.

## Conclusion

Our proposed definition and conceptual framework of TB vulnerability reaffirms that inequality in social determinants of health is an underlying contributor to TB vulnerability in populations. The proposed operational criteria for TB vulnerable populations are useful for developing interventions to address underlying social inequalities that contribute to increased TB exposure and risks, limit access to high quality TB health services, and differential outcomes related to the disease. Examining TB vulnerability from social determinants of health and equity perspectives aligns with the End TB strategy’s principles and strategic directions adopted by key global stakeholders working in TB, and gives us insights into how to improve TB care to achieve better health equity among the most vulnerable populations.

## Supplementary Material

Supplemental Material

## Data Availability

The dataset used and/or analysed during the current study are available from the corresponding author on reasonable request.
