# Supplementary material for: Defining tuberculosis vulnerability based on an adapted social determinants of health framework: a narrative review
Source: Glob Public Health. 2023 Jun 11;18(1):2221729. doi: 10.1080/17441692.2023.2221729 (PMC10259234; doi:10.1080/17441692.2023.2221729)
Supplement: Supplemental Material [file RGPH_A_2221729_SM9206.docx]

**Appendix 1: Literature Searches**

Ovid MEDLINE(R) <1946 to October Week 4 2021>

| **#** | **Searches** | **Results** | **Comment** |
| --- | --- | --- | --- |
| 1 | *tuberculosis/ or *latent tuberculosis/ or *tuberculosis, pleural/ or *tuberculosis, pulmonary/ or *Mycobacterium tuberculosis/ | 167917 | Tuberculosis terms - focus |
| 2 | (vulnerable or vulnerability).ti,ab,kf. | 125521 |  |
| 3 | (definition* or define* or criteria* or "key population" or "high priority" or "high risk" or "at risk" or "disproportionate*" or framework* or dynamic* or susceptibl* or factor or factors).ti,ab,kf. | 5386438 |  |
| 4 | risk/ or risk factors/ or morbidity/ or incidence/ or prevalence/ | 1430186 |  |
| 5 | or/2-4 | 6153192 | Definition or risk terms |
| 6 | Vulnerable Populations/ [****MeSH since 2003****] | 12057 |  |
| 7 | ("Foreign-born" or "foreign born").ti,ab,kf. | 3259 |  |
| 8 | disabled persons/ or disabled children/ or persons with mental disabilities/ or mentally ill persons/ or disaster victims/ or "emigrants and immigrants"/ or undocumented immigrants/ or enslaved persons/ or homeless persons/ or homeless youth/ or medically uninsured/ or prisoners/ or refugees/ or sex workers/ or "sexual and gender minorities"/ or intersex persons/ or transgender persons/ or "transients and migrants"/ or veterans/ [****Misc. marginalization terms****] | 159784 |  |
| 9 | (displaced adj2 (people* or person* or population* or internally)).ti,ab,kf. | 1261 |  |
| 10 | continental population groups/ or african continental ancestry group/ or african americans/ or american native continental ancestry group/ or indians, central american/ or indians, north american/ or alaskan natives/ or indigenous canadians/ or inuits/ or american natives/ or indians, south american/ or asian continental ancestry group/ or asian americans/ or european continental ancestry group/ or oceanic ancestry group/ or ethnic groups/ or amish/ or arabs/ or indigenous peoples/ or roma/ or hispanic americans/ or mexican americans/ or jews/ | 314142 | Ethnic group terms |
| 11 | (indigenous or aboriginal* or nomad* or (mobile adj2 population*)).ti,ab,kf. | 38662 |  |
| 12 | substance-related disorders/ or alcohol-related disorders/ or alcohol-induced disorders/ or exp alcohol-induced disorders, nervous system/ or cardiomyopathy, alcoholic/ or fetal alcohol spectrum disorders/ or exp liver diseases, alcoholic/ or pancreatitis, alcoholic/ or psychoses, alcoholic/ or alcoholic intoxication/ or alcoholism/ or binge drinking/ or amphetamine-related disorders/ or cocaine-related disorders/ or exp drug overdose/ or inhalant abuse/ or marijuana abuse/ or neonatal abstinence syndrome/ or phencyclidine abuse/ or psychoses, substance-induced/ or substance abuse, intravenous/ or substance abuse, oral/ or substance withdrawal syndrome/ or alcohol withdrawal delirium/ or alcohol withdrawal seizures/ or "tobacco use disorder"/ or drug user/ | 273844 | Addiction related terms |
| 13 | smoking/ or pipe smoking/ or water pipe smoking/ or smoking, non-tobacco products/ or cocaine smoking/ or marijuana smoking/ or tobacco smoking/ or cigar smoking/ or cigarette smoking/ or vaping/ | 154452 | Smoking terms expanded |
| 14 | narcotic-related disorders/ or opioid-related disorders/ or heroin dependence/ or morphine dependence/ or opiate overdose/ or opium dependence/ or substance abuse, oral/ or substance withdrawal syndrome/ or alcohol withdrawal delirium/ or alcohol withdrawal seizures/ or "tobacco use disorder"/ | 60746 | Narcotics/Opioid terms |
| 15 | (pwud or pwid).ti,ab,kf. | 2012 |  |
| 16 | socioeconomic factors/ or economic factors/ or economic status/ or educational status/ or academic failure/ or literacy/ or employment/ or career mobility/ or child labor/ or employment, supported/ or personnel downsizing/ or return to work/ or teleworking/ or unemployment/ or workplace/ | 292570 | Socioeconomic terms |
| 17 | income/ or pensions/ or employee retirement income security act/ or remuneration/ or "salaries and fringe benefits"/ or family leave/ or parental leave/ or health benefit plans, employee/ or sick leave/ | 66601 | Income terms |
| 18 | medical indigency/ or poverty/ or poverty areas/ or social change/ or social class/ or social mobility/ or social conditions/ | 113399 | Poverty terms |
| 19 | residence characteristics/ or catchment area, health/ or housing/ or housing for the elderly/ or public housing/ or independent living/ | 71565 | Residence terms |
| 20 | rural health/ or suburban health/ or urban health/ | 38622 |  |
| 21 | ((working or urban or rural) adj2 (poor or poverty)).ti,ab,kf. | 3924 |  |
| 22 | (slum adj2 dweller*).ti,ab,kf. | 249 |  |
| 23 | overcrowding.ti,ab,kf. | 2774 |  |
| 24 | family characteristics/ or marital status/ or divorce/ or marriage/ or single person/ or single parent/ or widowhood/ | 63213 | Family characteristics |
| 25 | (household* adj2 contact*).ti,ab,kf. | 2750 |  |
| 26 | health education/ or health literacy/ or health fairs/ | 69526 | Education or literacy terms |
| 27 | dehumanization/ or commodification/ or social adjustment/ or social conformity/ or social isolation/ or social marginalization/ or social stigma/ or stereotyping/ | 62992 | Social terms |
| 28 | attitude to health/ or health knowledge, attitudes, practice/ or "treatment adherence and compliance"/ or "patient acceptance of health care"/ or patient compliance/ or medication adherence/ or directly observed therapy/ or no-show patients/ or patient dropouts/ or patient participation/ or patient satisfaction/ or patient preference/ or treatment refusal/ or vaccination refusal/ | 434109 | Attitude, compliance and vaccination refusal terms |
| 29 | "Social Determinants of Health"/ | 4799 |  |
| 30 | (social adj2 determinant* adj2 (TB or tuberculosis)).ti,ab,kf. | 38 |  |
| 31 | minors/ or farmers/ or metal workers/ or miners/ | 5823 |  |
| 32 | (mines or miners or ((tea or coffee or sugar or plantation*) adj2 work*) or farmer* or ((industrial or factory or brick or cement or quarry or garment*) adj2 worker*)).ti,ab,kf. | 31958 |  |
| 33 | occupational exposure/ or maximum allowable concentration/ or "threshold limit values"/ or war exposure/ | 66163 | Occupational terms |
| 34 | malnutrition/ or deficiency diseases/ or avitaminosis/ or ascorbic acid deficiency/ or scurvy/ or vitamin a deficiency/ or vitamin b deficiency/ or choline deficiency/ or folic acid deficiency/ or hyperhomocysteinemia/ or pellagra/ or riboflavin deficiency/ or thiamine deficiency/ or beriberi/ or wernicke encephalopathy/ or vitamin b 6 deficiency/ or vitamin b 12 deficiency/ or anemia, pernicious/ or subacute combined degeneration/ or vitamin d deficiency/ or rickets/ or osteomalacia/ or "chronic kidney disease-mineral and bone disorder"/ or rickets, hypophosphatemic/ or familial hypophosphatemic rickets/ or vitamin e deficiency/ or steatitis/ or vitamin k deficiency/ or vitamin k deficiency bleeding/ or magnesium deficiency/ or potassium deficiency/ or protein deficiency/ or protein-energy malnutrition/ or swayback/ or severe acute malnutrition/ or kwashiorkor/ or starvation/ | 127345 | Nutrition terms |
| 35 | malnourish*.ti,ab,kf. | 10264 |  |
| 36 | hiv infections/ or acquired immunodeficiency syndrome/ or acute retroviral syndrome/ or aids arteritis, central nervous system/ or aids dementia complex/ or aids-associated nephropathy/ or aids-related complex/ or aids-related opportunistic infections/ or hiv enteropathy/ or hiv seropositivity/ or hiv wasting syndrome/ or hiv-associated lipodystrophy syndrome/ or HIV Seronegativity/ | 298558 | HIV terms |
| 37 | plhiv.ti,ab,kf. | 1314 |  |
| 38 | diabetes mellitus/ or diabetes mellitus, type 1/ or wolfram syndrome/ or diabetes mellitus, type 2/ or diabetes mellitus, lipoatrophic/ or diabetes, gestational/ or diabetic ketoacidosis/ or donohue syndrome/ or latent autoimmune diabetes in adults/ or prediabetic state/ | 347310 | Diabetes terms |
| 39 | Pregnant Women/ or postpartum period/ or lactation/ or milk ejection/ or pregnancy rate/ or pregnancy trimesters/ or pregnancy trimester, first/ or pregnancy trimester, second/ or pregnancy trimester, third/ or exp pregnancy/ or pregnancy, complicaitons, infectious/ or climacteric/ or andropause/ or menopause/ or menopause, premature/ or perimenopause/ or postmenopause/ or premenopause/ | 1022940 | Pregnancy, lactation, menopause terms |
| 40 | (*tuberculosis/ or *latent tuberculosis/ or *tuberculosis, pleural/ or *tuberculosis, pulmonary/) and treatment outcome/ [****Indexing for post TB sequelae****] | 4349 |  |
| 41 | ("post Tuberculosis sequela*" or "post-tuberculosis sequela*").ti,ab,kf. | 10 |  |
| 42 | or/6-41 | 3377048 | Vulnerable population terms |
| 43 | 1 and 5 and 42 | 11824 | Base clinical set 1 - TB and definition and vulnerable populations |
| 44 | limit 43 to (english or French or Chinese) | 10333 | Language limit |
| 45 | limit 44 to yr="2010 -Current" | 6340 | Publication date limit final results |
| 46 | (afghanistan or albania or algeria or american samoa or angola or "antigua and barbuda" or antigua or barbuda or argentina or armenia or armenian or aruba or azerbaijan or bahrain or bangladesh or barbados or republic of belarus or belarus or byelarus or belorussia or byelorussian or belize or british honduras or benin or dahomey or bhutan or bolivia or "bosnia and herzegovina" or bosnia or herzegovina or botswana or bechuanaland or brazil or brasil or bulgaria or burkina faso or burkina fasso or upper volta or burundi or urundi or cabo verde or cape verde or cambodia or kampuchea or khmer republic or cameroon or cameron or cameroun or central african republic or ubangi shari or chad or chile or china or colombia or comoros or comoro islands or iles comores or mayotte or democratic republic of the congo or democratic republic congo or congo or zaire or costa rica or "cote d’ivoire" or "cote d’ ivoire" or cote divoire or cote d ivoire or ivory coast or croatia or cuba or cyprus or czech republic or czechoslovakia or djibouti or french somaliland or dominica or dominican republic or ecuador or egypt or united arab republic or el salvador or equatorial guinea or spanish guinea or eritrea or estonia or eswatini or swaziland or ethiopia or fiji or gabon or gabonese republic or gambia or "georgia (republic)" or georgian or ghana or gold coast or gibraltar or greece or grenada or guam or guatemala or guinea or guinea bissau or guyana or british guiana or haiti or hispaniola or honduras or hungary or india or indonesia or timor or iran or iraq or isle of man or jamaica or jordan or kazakhstan or kazakh or kenya or "democratic people’s republic of korea" or republic of korea or north korea or south korea or korea or kosovo or kyrgyzstan or kirghizia or kirgizstan or kyrgyz republic or kirghiz or laos or lao pdr or "lao people's democratic republic" or latvia or lebanon or lebanese republic or lesotho or basutoland or liberia or libya or libyan arab jamahiriya or lithuania or macau or macao or republic of north macedonia or macedonia or madagascar or malagasy republic or malawi or nyasaland or malaysia or malay federation or malaya federation or maldives or indian ocean islands or indian ocean or mali or malta or micronesia or federated states of micronesia or kiribati or marshall islands or nauru or northern mariana islands or palau or tuvalu or mauritania or mauritius or mexico or moldova or moldovian or mongolia or montenegro or morocco or ifni or mozambique or portuguese east africa or myanmar or burma or namibia or nepal or netherlands antilles or nicaragua or niger or nigeria or oman or muscat or pakistan or panama or papua new guinea or new guinea or paraguay or peru or philippines or philipines or phillipines or phillippines or poland or "polish people's republic" or portugal or portuguese republic or puerto rico or romania or russia or russian federation or ussr or soviet union or union of soviet socialist republics or rwanda or ruanda or samoa or pacific islands or polynesia or samoan islands or navigator island or navigator islands or "sao tome and principe" or saudi arabia or senegal or serbia or seychelles or sierra leone or slovakia or slovak republic or slovenia or melanesia or solomon island or solomon islands or norfolk island or norfolk islands or somalia or south africa or south sudan or sri lanka or ceylon or "saint kitts and nevis" or "st. kitts and nevis" or saint lucia or "st. lucia" or "saint vincent and the grenadines" or saint vincent or "st. vincent" or grenadines or sudan or suriname or surinam or dutch guiana or netherlands guiana or syria or syrian arab republic or tajikistan or tadjikistan or tadzhikistan or tadzhik or tanzania or tanganyika or thailand or siam or timor leste or east timor or togo or togolese republic or tonga or "trinidad and tobago" or trinidad or tobago or tunisia or turkey or turkmenistan or turkmen or uganda or ukraine or uruguay or uzbekistan or uzbek or vanuatu or new hebrides or venezuela or vietnam or viet nam or middle east or west bank or gaza or palestine or yemen or yugoslavia or zambia or zimbabwe or northern rhodesia or global south or africa south of the sahara or sub-saharan africa or subsaharan africa or africa, central or central africa or africa, northern or north africa or northern africa or magreb or maghrib or sahara or africa, southern or southern africa or africa, eastern or east africa or eastern africa or africa, western or west africa or western africa or west indies or indian ocean islands or caribbean or central america or latin america or "south and central america" or south america or asia, central or central asia or asia, northern or north asia or northern asia or asia, southeastern or southeastern asia or south eastern asia or southeast asia or south east asia or asia, western or western asia or europe, eastern or east europe or eastern europe or developing country or developing countries or developing nation? or developing population? or developing world or less developed countr* or less developed nation? or less developed population? or less developed world or lesser developed countr* or lesser developed nation? or lesser developed population? or lesser developed world or under developed countr* or under developed nation? or under developed population? or under developed world or underdeveloped countr* or underdeveloped nation? or underdeveloped population? or underdeveloped world or middle income countr* or middle income nation? or middle income population? or low income countr* or low income nation? or low income population? or lower income countr* or lower income nation? or lower income population? or underserved countr* or underserved nation? or underserved population? or underserved world or under served countr* or under served nation? or under served population? or under served world or deprived countr* or deprived nation? or deprived population? or deprived world or poor countr* or poor nation? or poor population? or poor world or poorer countr* or poorer nation? or poorer population? or poorer world or developing econom* or less developed econom* or lesser developed econom* or under developed econom* or underdeveloped econom* or middle income econom* or low income econom* or lower income econom* or low gdp or low gnp or low gross domestic or low gross national or lower gdp or lower gnp or lower gross domestic or lower gross national or lmic or lmics or third world or lami countr* or transitional countr* or emerging economies or emerging nation?).ti,ab,sh,kf. | 1852391 | Cochrane – LMIC country terms |
| 47 | 45 and 46 | 3946 | LMIC specific results |
|  | Information only – Health personnel results |  |  |
| 48 | exp Health Personnel/ | 559260 |  |
| 49 | 1 and 5 and 48 [****Health Personnel results****] | 901 |  |
| 50 | limit 49 to (english language and yr="2010 -Current") | 462 |  |
| 51 | 50 not 45 [****Unique healthcare personnel results****] | 215 |  |
| 52 | 45 not 47 | 2292 |  |
|  | | |  |

Medline Epub Ahead of Print

Ovid MEDLINE(R) Epub Ahead of Print

| **#** | **Searches** | **Results** | **Comment** |
| --- | --- | --- | --- |
| 1 | (tuberculosis or tubercular).ti,ab,kf. | 1486 | Tuberculosis terms - focus |
| 2 | (vulnerable or vulnerability).ti,ab,kf. | 4217 |  |
| 3 | (definition* or define* or criteria* or "key population" or "high priority" or "high risk" or "at risk" or "disproportionate*" or framework* or dynamic* or susceptibl* or factor or factors).ti,ab,kf. | 109611 |  |
| 4 | (risk or risks or mobrid* or inciden* or prevalen*).ti,ab,kf. | 76499 |  |
| 5 | or/2-4 | 146398 | Definition or risk terms |
| 6 | (Vulnerable adj2 (population* or people* or patient*)).ti,ab,kf. | 853 |  |
| 7 | ("Foreign-born" or "foreign born").ti,ab,kf. | 112 |  |
| 8 | (((disabled or diasabilit*) adj2 (person* or adult* or child*)) or (mental* adj2 ill*) or (disaster adj2 victim*) or emigrant* or immigrant* or enslaved or homeless* or (medical* adj2 uninsured) or prisoner* or refugee* or (sex adj2 worker*) or prostitute* or ((sexual or gender) adj2 minorit*) or intersex or transgender* or transient* or migrant* or veteran*).ti,ab,kf. [****Misc. marginalization terms****] | 8699 |  |
| 9 | (displaced adj2 (people* or person* or population* or internally)).ti,ab,kf. | 36 |  |
| 10 | ((continental adj2 population adj2 group*) or ((african or asian or oceanic) adj2 ancestry) or (american adj2 native*) or ((south or central or north) adj2 american adj2 indian*) or (alaskan adj2 native*) or inuits or (first adj2 nations) or ((asian or chineses or korean or japanese or hispanic) adj2 (american* or canadian*)) or (european adj2 continental adj2 ancestry) or (ethnic adj2 group*) or amish or arab or arabs or arabic or roma or romas or mexican* or jews or jews or jewish).ti,ab,kf. | 2399 | Ethnic group terms |
| 11 | (indigenous or aboriginal* or nomad* or (mobile adj2 population*)).ti,ab,kf. | 931 |  |
| 12 | ((("substance-related" or "alcohol-related" or "alcohol-induced" or "amphetamine-related" or "cocaine-related" or tobacco) adj2 (disease* or disorder* or dependenc* or abuse or psychos* or seizure* or withdrawal)) or alcoholic* or intoxication or intoxicated or (fetal adj2 alcohol adj2 (disease* or disorder* or syndrome* or spectrum)) or pancreatitis or alcoholism or (binge adj2 drinking) or overdose* or overdosing or overdosed or addiction* or addicted or ((drug or substance or inhalant or marijuana or phencyclidine) adj2 (abuse or user*)) or (withdrawal adj2 (syndrome* or delirium or seizure*)) or (neonatal adj2 abstinence adj2 syndrome*)).ti,ab,kf. | 5156 | Addiction related terms |
| 13 | (smoking or smoker* or vaping).ti,ab,kf. | 4296 | Smoking terms expanded |
| 14 | ((narcotic or opioid or opiate* or opium or heroin or morphine or substance) adj2 (disease* or disorder* or dependenc* or abuse or psychos* or seizure* or withdrawal)).ti,ab,kf. | 1973 | Narcotics/Opioid terms |
| 15 | (pwud or pwid).ti,ab,kf. | 131 |  |
| 16 | (((socioeconomic or economic or education*) adj2 (factor* or status or class* or change* or mobility or condition*)) or (academic adj2 (failure or success* or achievement*)) or literacy or employment or (career adj2 mobility) or (child adj2 (labor or labour)) or employment or (personnel adj2 downsizing) or (return adj work) or teleworking or unemployment or workplace).ti,ab,kf. | 6424 | Socioeconomic terms |
| 17 | (income* or pension* or (retirement adj3 security) or remuneration or salary or salaries or (fringe adj2 benefit*) or ((family or parental or paternal or maternal or sick) adj2 leave*) or (health adj2 benefit adj2 plan*)).ti,ab,kf. | 4875 | Income terms |
| 18 | ((medical adj2 indigen*) or poverty or (social adj2 (change* or class* or mobility or condition*))).ti,ab,kf. | 1314 | Poverty terms |
| 19 | (((residence* or house* or home*) adj2 characteristic*) or (catchment adj2 area*) or housing or (independent* adj2 living)).ti,ab,kf. | 1040 | Residence terms |
| 20 | ((rural or suburban or urban) adj2 health).ti,ab,kf. | 301 |  |
| 21 | ((working or urban or rural) adj2 (poor or poverty)).ti,ab,kf. | 67 |  |
| 22 | (slum adj2 dweller*).ti,ab,kf. | 6 |  |
| 23 | overcrowding.ti,ab,kf. | 73 |  |
| 24 | ((family adj2 characteristic*) or (marital adj2 status) or divorce* or marriage* or (single adj2 (person* or parent*)) or widow*).ti,ab,kf. | 1166 | Family characteristics |
| 25 | (household* adj2 contact*).ti,ab,kf. | 53 |  |
| 26 | (health adj2 (educat* or fair*)).ti,ab,kf. [***Education or literacy terms****] | 1258 | Education or literacy terms |
| 27 | (dehumanization or commodification or (social adj2 (adjustment* or conformity or isolation or marginalization)) or stigma or stigmas or stigmatization or stereotyp*).ti,ab,kf. | 2519 | Social terms |
| 28 | ((attitude* adj2 health) or (health adj2 (knowledge* or practice*)) or ((patient* or treatment or medication) adj2 (adherence or compliance or acceptance or dropout or dropouts or satisfaction or preference* or refusal)) or (directly adj2 observed adj2 therap*) or "no-show patients" or (vaccin adj2 (hesitan* or refusal))).ti,ab,kf. | 3914 | Attitude, compliance and vaccination refusal terms |
| 29 | (social adj2 determinant* adj2 Health).ti,ab,kf. | 512 |  |
| 30 | (social adj2 determinant* adj2 (TB or tuberculosis)).ti,ab,kf. | 1 |  |
| 31 | (minor or minor*).ti,ab,kf. | 5484 |  |
| 32 | (mines or miner* or ((tea or coffee or sugar or plantation*) adj2 work*) or farmer* or ((industrial or factory or brick or cement or quarry or garment* or metal) adj2 worker*)).ti,ab,kf. | 3277 |  |
| 33 | (((occupation* or war or wars) adj2 exposure*) or (maximum adj2 allowable adj2 concentrat*) or (threshold adj2 limit adj2 value*)).ti,ab,kf. | 416 | Occupational terms |
| 34 | (malnutrition or malnourish* or (deficiency adj2 disease*) or avitaminosis or (ascorbic adj2 acid adj2 deficien*) or scurvy or ((vitamin* or choline or "folic acid" or riboflavin or thiamine or magnesium or potassium or protein* or mineral*) adj2 deficien*) or hyperhomocysteinemia or pellagra or beriberi or (wernicke adj2 encephalopath*) or anemia* or anaemia* or anemic or anaemic or (subacute adj2 combined adj2 degenerat*) or rickets or osteomalacia* or oesteomalacia* or (mineral adj2 bone adj2 (disease* or disorder*)) or rickets or steatitis or swayback or kwashiorkor or starvation or starving).ti,ab,kf. | 3649 | Nutrition terms |
| 35 | (hiv or (acquired adj2 (immunodeficiency or "immune deficiency") adj2 syndrome*) or (acute adj2 retroviral adj2 syndrome*) or (aids adj2 (arteritis or demetia or nephropath* or complex or infection*))).ti,ab,kf. [****HIV terms****] | 3855 | HIV terms |
| 36 | plhiv.ti,ab,kf. | 75 |  |
| 37 | (diabetes or diabetic* or prediabetic* or ((wolfram or donohue) adj2 syndrome*)).ti,ab,kf. [****Diabetes terms****] | 11002 | Diabetes terms |
| 38 | (Pregnant or pregnancy or pregnancies or postpartum or lactation or lactatiing or climacteric or andropaus* or menopaus* or perimenopaus* or postmenopaus* or premenopaus*).ti,ab,kf. [**** Pregnancy, lactation, menopause terms****] | 10484 | Pregnancy, lactation, menopause terms |
| 39 | ("post Tuberculosis sequela*" or "post-tuberculosis sequela*").ti,ab,kf. | 0 |  |
| 40 | or/6-39 | 67059 | vulnerable population terms |
| 41 | 1 and 5 and 40 [****Base clinical set 1 - TB and definition and vulnerable populations****] | 295 | Base clinical set 1 - TB and definition and vulnerable populations |
| 42 | limit 41 to (english or French or Chinese) | 293 | Language limit |
| 43 | limit 42 to yr="2010 -Current" | 278 | Publication year limit – Final results |
| 44 | (afghanistan or albania or algeria or american samoa or angola or "antigua and barbuda" or antigua or barbuda or argentina or armenia or armenian or aruba or azerbaijan or bahrain or bangladesh or barbados or republic of belarus or belarus or byelarus or belorussia or byelorussian or belize or british honduras or benin or dahomey or bhutan or bolivia or "bosnia and herzegovina" or bosnia or herzegovina or botswana or bechuanaland or brazil or brasil or bulgaria or burkina faso or burkina fasso or upper volta or burundi or urundi or cabo verde or cape verde or cambodia or kampuchea or khmer republic or cameroon or cameron or cameroun or central african republic or ubangi shari or chad or chile or china or colombia or comoros or comoro islands or iles comores or mayotte or democratic republic of the congo or democratic republic congo or congo or zaire or costa rica or "cote d’ivoire" or "cote d’ ivoire" or cote divoire or cote d ivoire or ivory coast or croatia or cuba or cyprus or czech republic or czechoslovakia or djibouti or french somaliland or dominica or dominican republic or ecuador or egypt or united arab republic or el salvador or equatorial guinea or spanish guinea or eritrea or estonia or eswatini or swaziland or ethiopia or fiji or gabon or gabonese republic or gambia or "georgia (republic)" or georgian or ghana or gold coast or gibraltar or greece or grenada or guam or guatemala or guinea or guinea bissau or guyana or british guiana or haiti or hispaniola or honduras or hungary or india or indonesia or timor or iran or iraq or isle of man or jamaica or jordan or kazakhstan or kazakh or kenya or "democratic people’s republic of korea" or republic of korea or north korea or south korea or korea or kosovo or kyrgyzstan or kirghizia or kirgizstan or kyrgyz republic or kirghiz or laos or lao pdr or "lao people's democratic republic" or latvia or lebanon or lebanese republic or lesotho or basutoland or liberia or libya or libyan arab jamahiriya or lithuania or macau or macao or republic of north macedonia or macedonia or madagascar or malagasy republic or malawi or nyasaland or malaysia or malay federation or malaya federation or maldives or indian ocean islands or indian ocean or mali or malta or micronesia or federated states of micronesia or kiribati or marshall islands or nauru or northern mariana islands or palau or tuvalu or mauritania or mauritius or mexico or moldova or moldovian or mongolia or montenegro or morocco or ifni or mozambique or portuguese east africa or myanmar or burma or namibia or nepal or netherlands antilles or nicaragua or niger or nigeria or oman or muscat or pakistan or panama or papua new guinea or new guinea or paraguay or peru or philippines or philipines or phillipines or phillippines or poland or "polish people's republic" or portugal or portuguese republic or puerto rico or romania or russia or russian federation or ussr or soviet union or union of soviet socialist republics or rwanda or ruanda or samoa or pacific islands or polynesia or samoan islands or navigator island or navigator islands or "sao tome and principe" or saudi arabia or senegal or serbia or seychelles or sierra leone or slovakia or slovak republic or slovenia or melanesia or solomon island or solomon islands or norfolk island or norfolk islands or somalia or south africa or south sudan or sri lanka or ceylon or "saint kitts and nevis" or "st. kitts and nevis" or saint lucia or "st. lucia" or "saint vincent and the grenadines" or saint vincent or "st. vincent" or grenadines or sudan or suriname or surinam or dutch guiana or netherlands guiana or syria or syrian arab republic or tajikistan or tadjikistan or tadzhikistan or tadzhik or tanzania or tanganyika or thailand or siam or timor leste or east timor or togo or togolese republic or tonga or "trinidad and tobago" or trinidad or tobago or tunisia or turkey or turkmenistan or turkmen or uganda or ukraine or uruguay or uzbekistan or uzbek or vanuatu or new hebrides or venezuela or vietnam or viet nam or middle east or west bank or gaza or palestine or yemen or yugoslavia or zambia or zimbabwe or northern rhodesia or global south or africa south of the sahara or sub-saharan africa or subsaharan africa or africa, central or central africa or africa, northern or north africa or northern africa or magreb or maghrib or sahara or africa, southern or southern africa or africa, eastern or east africa or eastern africa or africa, western or west africa or western africa or west indies or indian ocean islands or caribbean or central america or latin america or "south and central america" or south america or asia, central or central asia or asia, northern or north asia or northern asia or asia, southeastern or southeastern asia or south eastern asia or southeast asia or south east asia or asia, western or western asia or europe, eastern or east europe or eastern europe or developing country or developing countries or developing nation? or developing population? or developing world or less developed countr* or less developed nation? or less developed population? or less developed world or lesser developed countr* or lesser developed nation? or lesser developed population? or lesser developed world or under developed countr* or under developed nation? or under developed population? or under developed world or underdeveloped countr* or underdeveloped nation? or underdeveloped population? or underdeveloped world or middle income countr* or middle income nation? or middle income population? or low income countr* or low income nation? or low income population? or lower income countr* or lower income nation? or lower income population? or underserved countr* or underserved nation? or underserved population? or underserved world or under served countr* or under served nation? or under served population? or under served world or deprived countr* or deprived nation? or deprived population? or deprived world or poor countr* or poor nation? or poor population? or poor world or poorer countr* or poorer nation? or poorer population? or poorer world or developing econom* or less developed econom* or lesser developed econom* or under developed econom* or underdeveloped econom* or middle income econom* or low income econom* or lower income econom* or low gdp or low gnp or low gross domestic or low gross national or lower gdp or lower gnp or lower gross domestic or lower gross national or lmic or lmics or third world or lami countr* or transitional countr* or emerging economies or emerging nation?).ti,ab,sh,kf. | 26198 | Cochrane - LMIC search terms |
| 45 | 43 and 44 [****LMIC specific results****] | 142 | Cochrane – LMIC results |

Medline-in-Process & In-Data Review

Ovid MEDLINE(R) In-Process & In-Data-Review Citations <1946 to October 27, 2021>

| **#** | **Searches** | **Results** | **Type** |
| --- | --- | --- | --- |
| 1 | (tuberculosis or tubercular).ti,ab,kf. | 1505 | Tuberculosis terms - focus |
| 2 | (vulnerable or vulnerability).ti,ab,kf. | 2899 |  |
| 3 | (definition* or define* or criteria* or "key population" or "high priority" or "high risk" or "at risk" or "disproportionate*" or framework* or dynamic* or susceptibl* or factor or factors).ti,ab,kf. | 91711 |  |
| 4 | (risk or risks or mobrid* or inciden* or prevalen*).ti,ab,kf. | 58784 |  |
| 5 | or/2-4 | 118947 | Definition or risk terms |
| 6 | (Vulnerable adj2 (population* or people* or patient*)).ti,ab,kf. | 512 |  |
| 7 | ("Foreign-born" or "foreign born").ti,ab,kf. | 42 |  |
| 8 | (((disabled or diasabilit*) adj2 (person* or adult* or child*)) or (mental* adj2 ill*) or (disaster adj2 victim*) or emigrant* or immigrant* or enslaved or homeless* or (medical* adj2 uninsured) or prisoner* or refugee* or (sex adj2 worker*) or prostitute* or ((sexual or gender) adj2 minorit*) or intersex or transgender* or transient* or migrant* or veteran*).ti,ab,kf. [****Misc. marginalization terms****] | 5916 |  |
| 9 | (displaced adj2 (people* or person* or population* or internally)).ti,ab,kf. | 17 |  |
| 10 | ((continental adj2 population adj2 group*) or ((african or asian or oceanic) adj2 ancestry) or (american adj2 native*) or ((south or central or north) adj2 american adj2 indian*) or (alaskan adj2 native*) or inuits or (first adj2 nations) or ((asian or chineses or korean or japanese or hispanic) adj2 (american* or canadian*)) or (european adj2 continental adj2 ancestry) or (ethnic adj2 group*) or amish or arab or arabs or arabic or roma or romas or mexican* or jews or jews or jewish).ti,ab,kf. | 1618 | Ethnic group terms |
| 11 | (indigenous or aboriginal* or nomad* or (mobile adj2 population*)).ti,ab,kf. | 630 |  |
| 12 | ((("substance-related" or "alcohol-related" or "alcohol-induced" or "amphetamine-related" or "cocaine-related" or tobacco) adj2 (disease* or disorder* or dependenc* or abuse or psychos* or seizure* or withdrawal)) or alcoholic* or intoxication or intoxicated or (fetal adj2 alcohol adj2 (disease* or disorder* or syndrome* or spectrum)) or pancreatitis or alcoholism or (binge adj2 drinking) or overdose* or overdosing or overdosed or addiction* or addicted or ((drug or substance or inhalant or marijuana or phencyclidine) adj2 (abuse or user*)) or (withdrawal adj2 (syndrome* or delirium or seizure*)) or (neonatal adj2 abstinence adj2 syndrome*)).ti,ab,kf. | 3952 | Addiction related terms |
| 13 | (smoking or smoker* or vaping).ti,ab,kf. | 3507 | Smoking terms expanded |
| 14 | ((narcotic or opioid or opiate* or opium or heroin or morphine or substance) adj2 (disease* or disorder* or dependenc* or abuse or psychos* or seizure* or withdrawal)).ti,ab,kf. | 1104 | Narcotics/Opioid terms |
| 15 | (pwud or pwid).ti,ab,kf. | 86 |  |
| 16 | (((socioeconomic or economic or education*) adj2 (factor* or status or class* or change* or mobility or condition*)) or (academic adj2 (failure or success* or achievement*)) or literacy or employment or (career adj2 mobility) or (child adj2 (labor or labour)) or employment or (personnel adj2 downsizing) or (return adj work) or teleworking or unemployment or workplace).ti,ab,kf. | 3559 | Socioeconomic terms |
| 17 | (income* or pension* or (retirement adj3 security) or remuneration or salary or salaries or (fringe adj2 benefit*) or ((family or parental or paternal or maternal or sick) adj2 leave*) or (health adj2 benefit adj2 plan*)).ti,ab,kf. | 3058 | Income terms |
| 18 | ((medical adj2 indigen*) or poverty or (social adj2 (change* or class* or mobility or condition*))).ti,ab,kf. | 704 | Poverty terms |
| 19 | (((residence* or house* or home*) adj2 characteristic*) or (catchment adj2 area*) or housing or (independent* adj2 living)).ti,ab,kf. | 596 | Residence terms |
| 20 | ((rural or suburban or urban) adj2 health).ti,ab,kf. | 208 |  |
| 21 | ((working or urban or rural) adj2 (poor or poverty)).ti,ab,kf. | 55 |  |
| 22 | (slum adj2 dweller*).ti,ab,kf. | 3 |  |
| 23 | overcrowding.ti,ab,kf. | 35 |  |
| 24 | ((family adj2 characteristic*) or (marital adj2 status) or divorce* or marriage* or (single adj2 (person* or parent*)) or widow*).ti,ab,kf. | 675 | Family characteristics |
| 25 | (household* adj2 contact*).ti,ab,kf. | 28 |  |
| 26 | (health adj2 (educat* or fair*)).ti,ab,kf. | 642 | Education or literacy terms |
| 27 | (dehumanization or commodification or (social adj2 (adjustment* or conformity or isolation or marginalization)) or stigma or stigmas or stigmatization or stereotyp*).ti,ab,kf. | 1275 | Social terms |
| 28 | ((attitude* adj2 health) or (health adj2 (knowledge* or practice*)) or ((patient* or treatment or medication) adj2 (adherence or compliance or acceptance or dropout or dropouts or satisfaction or preference* or refusal)) or (directly adj2 observed adj2 therap*) or "no-show patients" or (vaccin adj2 (hesitan* or refusal))).ti,ab,kf. | 2357 | Attitude, compliance and vaccination refusal terms |
| 29 | (social adj2 determinant* adj2 Health).ti,ab,kf. | 287 |  |
| 30 | (social adj2 determinant* adj2 (TB or tuberculosis)).ti,ab,kf. | 0 |  |
| 31 | (minor or minor*).ti,ab,kf. | 3403 |  |
| 32 | (mines or miner* or ((tea or coffee or sugar or plantation*) adj2 work*) or farmer* or ((industrial or factory or brick or cement or quarry or garment* or metal) adj2 worker*)).ti,ab,kf. | 2356 |  |
| 33 | (((occupation* or war or wars) adj2 exposure*) or (maximum adj2 allowable adj2 concentrat*) or (threshold adj2 limit adj2 value*)).ti,ab,kf. | 244 | Occupational terms |
| 34 | (malnutrition or malnourish* or (deficiency adj2 disease*) or avitaminosis or (ascorbic adj2 acid adj2 deficien*) or scurvy or ((vitamin* or choline or "folic acid" or riboflavin or thiamine or magnesium or potassium or protein* or mineral*) adj2 deficien*) or hyperhomocysteinemia or pellagra or beriberi or (wernicke adj2 encephalopath*) or anemia* or anaemia* or anemic or anaemic or (subacute adj2 combined adj2 degenerat*) or rickets or osteomalacia* or oesteomalacia* or (mineral adj2 bone adj2 (disease* or disorder*)) or rickets or steatitis or swayback or kwashiorkor or starvation or starving).ti,ab,kf. | 3269 | Nutrition terms |
| 35 | (hiv or (acquired adj2 (immunodeficiency or "immune deficiency") adj2 syndrome*) or (acute adj2 retroviral adj2 syndrome*) or (aids adj2 (arteritis or demetia or nephropath* or complex or infection*))).ti,ab,kf. | 2880 | HIV terms |
| 36 | plhiv.ti,ab,kf. | 65 |  |
| 37 | (diabetes or diabetic* or prediabetic* or ((wolfram or donohue) adj2 syndrome*)).ti,ab,kf. | 11806 | Diabetes terms |
| 38 | (Pregnant or pregnancy or pregnancies or postpartum or lactation or lactatiing or climacteric or andropaus* or menopaus* or perimenopaus* or postmenopaus* or premenopaus*).ti,ab,kf. | 8778 | Pregnancy, lactation, menopause terms |
| 39 | ("post Tuberculosis sequela*" or "post-tuberculosis sequela*").ti,ab,kf. | 0 |  |
| 40 | or/6-39 | 51016 | vulnerable population terms |
| 41 | 1 and 5 and 40 | 281 | Base clinical set 1 - TB and definition and vulnerable populations |
| 42 | limit 41 to (english or french or chinese) | 278 | Language limit |
| 43 | limit 42 to yr="2010 -Current" | 278 | Publication year limit – Final results |
| 44 | (afghanistan or albania or algeria or american samoa or angola or "antigua and barbuda" or antigua or barbuda or argentina or armenia or armenian or aruba or azerbaijan or bahrain or bangladesh or barbados or republic of belarus or belarus or byelarus or belorussia or byelorussian or belize or british honduras or benin or dahomey or bhutan or bolivia or "bosnia and herzegovina" or bosnia or herzegovina or botswana or bechuanaland or brazil or brasil or bulgaria or burkina faso or burkina fasso or upper volta or burundi or urundi or cabo verde or cape verde or cambodia or kampuchea or khmer republic or cameroon or cameron or cameroun or central african republic or ubangi shari or chad or chile or china or colombia or comoros or comoro islands or iles comores or mayotte or democratic republic of the congo or democratic republic congo or congo or zaire or costa rica or "cote d’ivoire" or "cote d’ ivoire" or cote divoire or cote d ivoire or ivory coast or croatia or cuba or cyprus or czech republic or czechoslovakia or djibouti or french somaliland or dominica or dominican republic or ecuador or egypt or united arab republic or el salvador or equatorial guinea or spanish guinea or eritrea or estonia or eswatini or swaziland or ethiopia or fiji or gabon or gabonese republic or gambia or "georgia (republic)" or georgian or ghana or gold coast or gibraltar or greece or grenada or guam or guatemala or guinea or guinea bissau or guyana or british guiana or haiti or hispaniola or honduras or hungary or india or indonesia or timor or iran or iraq or isle of man or jamaica or jordan or kazakhstan or kazakh or kenya or "democratic people’s republic of korea" or republic of korea or north korea or south korea or korea or kosovo or kyrgyzstan or kirghizia or kirgizstan or kyrgyz republic or kirghiz or laos or lao pdr or "lao people's democratic republic" or latvia or lebanon or lebanese republic or lesotho or basutoland or liberia or libya or libyan arab jamahiriya or lithuania or macau or macao or republic of north macedonia or macedonia or madagascar or malagasy republic or malawi or nyasaland or malaysia or malay federation or malaya federation or maldives or indian ocean islands or indian ocean or mali or malta or micronesia or federated states of micronesia or kiribati or marshall islands or nauru or northern mariana islands or palau or tuvalu or mauritania or mauritius or mexico or moldova or moldovian or mongolia or montenegro or morocco or ifni or mozambique or portuguese east africa or myanmar or burma or namibia or nepal or netherlands antilles or nicaragua or niger or nigeria or oman or muscat or pakistan or panama or papua new guinea or new guinea or paraguay or peru or philippines or philipines or phillipines or phillippines or poland or "polish people's republic" or portugal or portuguese republic or puerto rico or romania or russia or russian federation or ussr or soviet union or union of soviet socialist republics or rwanda or ruanda or samoa or pacific islands or polynesia or samoan islands or navigator island or navigator islands or "sao tome and principe" or saudi arabia or senegal or serbia or seychelles or sierra leone or slovakia or slovak republic or slovenia or melanesia or solomon island or solomon islands or norfolk island or norfolk islands or somalia or south africa or south sudan or sri lanka or ceylon or "saint kitts and nevis" or "st. kitts and nevis" or saint lucia or "st. lucia" or "saint vincent and the grenadines" or saint vincent or "st. vincent" or grenadines or sudan or suriname or surinam or dutch guiana or netherlands guiana or syria or syrian arab republic or tajikistan or tadjikistan or tadzhikistan or tadzhik or tanzania or tanganyika or thailand or siam or timor leste or east timor or togo or togolese republic or tonga or "trinidad and tobago" or trinidad or tobago or tunisia or turkey or turkmenistan or turkmen or uganda or ukraine or uruguay or uzbekistan or uzbek or vanuatu or new hebrides or venezuela or vietnam or viet nam or middle east or west bank or gaza or palestine or yemen or yugoslavia or zambia or zimbabwe or northern rhodesia or global south or africa south of the sahara or sub-saharan africa or subsaharan africa or africa, central or central africa or africa, northern or north africa or northern africa or magreb or maghrib or sahara or africa, southern or southern africa or africa, eastern or east africa or eastern africa or africa, western or west africa or western africa or west indies or indian ocean islands or caribbean or central america or latin america or "south and central america" or south america or asia, central or central asia or asia, northern or north asia or northern asia or asia, southeastern or southeastern asia or south eastern asia or southeast asia or south east asia or asia, western or western asia or europe, eastern or east europe or eastern europe or developing country or developing countries or developing nation? or developing population? or developing world or less developed countr* or less developed nation? or less developed population? or less developed world or lesser developed countr* or lesser developed nation? or lesser developed population? or lesser developed world or under developed countr* or under developed nation? or under developed population? or under developed world or underdeveloped countr* or underdeveloped nation? or underdeveloped population? or underdeveloped world or middle income countr* or middle income nation? or middle income population? or low income countr* or low income nation? or low income population? or lower income countr* or lower income nation? or lower income population? or underserved countr* or underserved nation? or underserved population? or underserved world or under served countr* or under served nation? or under served population? or under served world or deprived countr* or deprived nation? or deprived population? or deprived world or poor countr* or poor nation? or poor population? or poor world or poorer countr* or poorer nation? or poorer population? or poorer world or developing econom* or less developed econom* or lesser developed econom* or under developed econom* or underdeveloped econom* or middle income econom* or low income econom* or lower income econom* or low gdp or low gnp or low gross domestic or low gross national or lower gdp or lower gnp or lower gross domestic or lower gross national or lmic or lmics or third world or lami countr* or transitional countr* or emerging economies or emerging nation?).ti,ab,sh,kf. [****Cochrane - LMIC search terms****] | 19402 | Cochrane - LMIC search terms |
| 45 | 43 and 44 | 154 | Cochrane – LMIC results |

EMBASE

Embase <1980 to 2021 Week 42>

| **#** | **Searches** | **Results** | **Type** |
| --- | --- | --- | --- |
| 1 | *tuberculosis/ or *latent tuberculosis/ or *tuberculous pleurisy/ or *lung tuberculosis/ or *Mycobacterium tuberculosis/ | 110528 | Tuberculosis terms - focus |
| 2 | (vulnerable or vulnerability).ti,ab,kf. | 192231 |  |
| 3 | (definition* or define* or criteria* or "key population" or "high priority" or "high risk" or "at risk" or "disproportionate*" or framework* or dynamic* or susceptibl* or factor or factors).ti,ab,kf. | 8195947 |  |
| 4 | risk/ or exp risk factor/ or morbidity/ or incidence/ or prevalence/ | 2780300 |  |
| 5 | or/2-4 | 9610378 | Definition or risk terms |
| 6 | Vulnerable Population/ | 21528 |  |
| 7 | ("Foreign-born" or "foreign born").ti,ab,kf. | 4410 |  |
| 8 | disabled person/ or handicapped child/ or mentally disabled person/ or physically disabled person/ or disaster victim/ or migrant/ or emigrant/ or exp forced migrant/ or immigrant/ or migrant worker/ or forced migrant/ or internally displaced person/ or undocumented immigrant/ or slave/ or homeless person/ or homeless man/ or homeless woman/ or homeless youth/ or homelessness/ or medically uninsured/ or prisoner/ or refugee/ or refugee camp/ or refugee crisis/ or humanitarian crisis/ or sex worker/ or "sexual and gender minority"/ or lgbtqia+ people/ or asexual people/ or intersex/ or lgbt people/ or bisexual female/ or bisexual male/ or homosexual female/ or homosexual male/ or transgender/ or "female to male transgender"/ or "male to female transgender"/ or men who have sex with men/ or "men who have sex with men and women"/ or women who have sex with women/ or "women who have sex with women and men"/ or veteran/ or transient*.ti,ab,kf. [****Misc. marginalization terms****] | 594574 |  |
| 9 | (displaced adj2 (people* or person* or population* or internally)).ti,ab,kf. | 1651 |  |
| 10 | ancestry group/ or asian american/ or asian continental ancestry group/ or australoid/ or black person/ or african american/ or african brazilian/ or african caribbean/ or british asian/ or caucasian/ or european american/ or hispanic/ or mexican american/ or indigenous people/ or alaska native/ or american indian/ or canadian aboriginal/ or first nation/ or indigenous australian/ or taiwanese aborigine/ or mongoloid/ or multiracial person/ or colored person/ or "creole (people)"/ or surinamese creole/ or mestizo/ or metis/ or mulatto/ or oceanic ancestry group/ or pacific islander/ or torres strait islander/ or exp ethnic group/ [****ethnic group terms - term ethnic group contains Amish, Roma etc. listed under the explode****] | 441630 | Ethnic group terms |
| 11 | (indigenous or aboriginal* or nomad* or (mobile adj2 population*)).ti,ab,kf. | 53699 |  |
| 12 | drug dependence/ or alcoholism/ or intoxication/ or alcohol intoxication/ or alcoholic cardiomyopathy/ or alcohol liver disease/ or alcohol liver cirrhosis/ or exp alcoholic fatty liver/ or exp alcoholic hepatitis/ or alcoholic pancreatitis/ or fetal alcohol syndrome/ or alcoholic pancreatitis/ or alcohol psychosis/ or korsakoff psychosis/ or wernicke korsakoff syndrome/ or alcohol abuse/ or binge drinking/ or heavy drinking/ or alcohol withdrawal syndrome/ or alcohol withdrawal seizure/ or alcoholic delirium/ or alcoholic hallucinosis/ or delirium tremens/ or alcoholic delirium/ or drug craving/ or neonatal abstinence syndrome/ or phencyclidine dependence/ or withdrawal seizure/ or alcohol withdrawal seizure/ or fetal alcohol syndrome/ or amphetamine dependence/ or benzodiazepine dependence/ or cannabis addiction/ or cocaine dependence/ or congenital drug dependence/ or drug abuse pattern/ or drug craving/ or drug misuse/ or drug seeking behavior/ or glue sniffing/ or methamphetamine dependence/ or multiple drug abuse/ or tobacco dependence/ | 416751 | Addiction related terms |
| 13 | "smoking and smoking related phenomena"/ or cannabis smoking/ or cocaine smoking/ or pipe smoking/ or water pipe smoking/ or smoking/ or cigar smoking/ or cigarette smoking/ or smoking habit/ or tobacco smoke/ or vaping/ | 406861 | Smoking terms expanded |
| 14 | narcotic dependence/ or heroin dependence/ or morphine addiction/ or opiate addiction/ or substance abuse/ or inhalant abuse/ or drug intoxication/ or drug overdose/ or opiate overdose/ or withdrawal syndrome/ or alcohol withdrawal syndrome/ or alcohol withdrawal seizure/ or alcoholic delirium/ or alcoholic hallucinosis/ or delirium tremens/ or alcoholic delirium/ or drug craving/ or neonatal abstinence syndrome/ or withdrawal seizure/ or alcohol withdrawal seizure/ | 153695 | Narcotics/Opioid terms |
| 15 | (pwud or pwid).ti,ab,kf. | 3455 |  |
| 16 | socioeconomics/ or economic status/ or household economic status/ or educational status/ or literacy/ or income group/ or highest income group/ or lowest income group/ or working poor/ or middle income group/ or employment/ or employment status/ or neet status/ or unemployment/ or full time employment/ or parttime employment/ or permanent employment/ or self employment/ or sheltered employment/ or supported employment/ or temporary employment/ or career mobility/ or child labor/ or personnel management/ or work/ or return to work/ or telecommuting/ or "work from home"/ or workplace/ | 471973 | Socioeconomic terms |
| 17 | Income/ or household income/ or family income/ or personal income/ or income security/ or "salary and fringe benefit"/ or family leave/ or parental leave/ or medical leave/ or pension/ or sabbatical/ or salary/ or health insurance/ | 226240 | Income terms |
| 18 | indigent/ or poverty/ or social change/ or social class/ or social status/ [*****Social mobility/ or social conditions/ indexed under social status****] | 181085 | Poverty terms |
| 19 | population structure/ or demography/ or population density/ or catchment area/ or "catchment area (health)"/ or hospital catchment area/ or residential care/ or housing/ or independent living/ [****Residence characteristics indexed under Demography****] | 380455 | Residence terms |
| 20 | (((public or elderly) adj2 housing) or (residence adj2 characteristic*)).ti,ab,kf. | 1695 |  |
| 21 | rural health/ or urban health/ or (suburban adj2 health).ti,ab,kf. | 2773 |  |
| 22 | working poor/ or ((working or urban or rural) adj2 (poor or poverty)).ti,ab,kf. | 4897 |  |
| 23 | (slum adj2 dweller*).ti,ab,kf. | 331 |  |
| 24 | "crowding (area)"/ or overcrowding.ti,ab,kf. | 7111 |  |
| 25 | "homemaking and the family"/ or civil union/ or cohabitation/ or marriage/ or consanguineous marriage/ or divorce/ or forced marriage/ or same-sex marriage/ or single-parent family/ or stepfamily/ or stepchild/ or stepparent/ or stepfather/ or stepmother/ or widowed person/ or widow/ or widower/ | 75770 | Family characteristics |
| 26 | (household* adj2 contact*).ti,ab,kf. | 3555 |  |
| 27 | health education/ or health literacy/ or ehealth literacy/ [****Education or literacy terms****] | 108097 |  |
| 28 | dehumanization/ or commodification/ or social adaptation/ or adjustment/ or social psychology/ or social isolation/ or social exclusion/ or ostracism/ or stigma/ or social stigma/ or stereotyping/ [****social conformity indexed under social psychology - social marginalization indexed under social exclusion/ or ostracism/****] | 157650 | Social terms |
| 29 | attitude to health/ or patient attitude/ or patient compliance/ or patient dropout/ or patient engagement/ or patient participation/ or patient preference/ or patient satisfaction/ or refusal to participate/ or medication compliance/ or treatment refusal/ or vaccination refusal/ or vaccine hesitancy/ [****health knowledge, attitudes, practice indexed and treatment adherence and compliance indexed under under attitude to health ---- Attitude, compliance and vaccination refusal terms*****] | 535935 | Attitude, compliance and vaccination refusal terms |
| 30 | "social determinants of health"/ | 11593 |  |
| 31 | (social adj2 determinant* adj2 (TB or tuberculosis)).ti,ab,kf. | 51 |  |
| 32 | "minor (person)"/ or farmers/ or metal workers/ or miners/ | 24991 |  |
| 33 | (mines or miners or ((tea or coffee or sugar or plantation*) adj2 work*) or farmer* or ((industrial or factory or brick or cement or quarry or garment*) adj2 worker*)).ti,ab,kf. | 41346 |  |
| 34 | exposure/ or occupational exposure/ or maximum allowable concentration/ or "threshold limit value"/ or war exposure/ | 263206 | Occupational terms |
| 35 | malnutrition/ or cachexia/ or protein deficiency/ or nutritional deficiency/ or protein deficiency/ or kwashiorkor/ or marasmus/ or protein c deficiency/ or protein calorie malnutrition/ or protein s deficiency/ or amino acid deficiency/ or arginine deficiency/ or glutamine deficiency/ or methionine deficiency/ or tryptophan deficiency/ or pellagra/ or choline deficiency/ or essential fatty acid deficiency/ or mineral deficiency/ or boron deficiency/ or calcium deficiency/ or hypocalcemia/ or chromium deficiency/ or copper deficiency/ or hypocupremia/ or iodine deficiency/ or iron deficiency/ or hypoferremia/ or ron deficiency anemia/ or iron refractory iron deficiency anemia/ or plummer vinson syndrome/ or magnesium deficiency/ or hypomagnesemia/ or manganese deficiency/ or molybdenum deficiency/ or nitrogen deficiency/ or phosphate deficiency/ or potassium deficiency/ or hypokalemia/ or selenium deficiency/ or keshan disease/ or sodium deficiency/ or hyponatremia/ or hypovolemic hyponatremia/ or potomania/ or zinc deficiency/ or taurine deficiency/ or vitamin deficiency/ or alpha tocopherol deficiency/ or ascorbic acid deficiency/ or scurvy/ or retinol deficiency/ or keratomalacia/ or vitamin b deficiency/ or b12 deficiency/ or pernicious anemia/ or biotin deficiency/ or folic acid deficiency/ or folate deficiency anemia/ or nicotinic acid deficiency/ or pantothenic acid deficiency/ or pyridoxine deficiency/ or riboflavin deficiency/ or thiamine deficiency/ or beriberi/ or vitamin d deficiency/ or vitamin k deficiency/ or starvation/ [****Nutrition terms****] | 309499 | Nutrition terms |
| 36 | malnourish*.ti,ab,kf. | 14839 |  |
| 37 | human immunodeficiency virus infection/ or acquired immune deficiency syndrome/ or aids related complex/ or acute hiv infection/ or aids arteritis/ or hiv associated dementia/ or hiv associated lipodystrophy/ or hiv associated nephropathy/ or hiv enteropathy/ or human immunodeficiency virus 1 infection/ or human immunodeficiency virus 2 infection/ or (hiv adj2 (seronegativ* or seropositiv*)).ti,ab,kf. | 394953 | HIV terms |
| 38 | plhiv.ti,ab,kf. | 2360 |  |
| 39 | diabetes mellitus/ or insulin dependent diabetes mellitus/ or latent autoimmune diabetes in adults/ or lipoatrophic diabetes mellitus/ or non insulin dependent diabetes mellitus/ or wolfram syndrome/ or pregnancy diabetes mellitus/ or maternal diabetes mellitus/ or diabetic ketoacidosis/ or leprechaunism/ or impaired glucose tolerance/ [****Diabetes terms ------- Donohue syndrome indexed under leprechaunism and prediabetic state indexed under impaired glucose tolerance****] | 947130 | Diabetes terms |
| 40 | named groups by pregnancy/ or multigravida/ or multipara/ or nulligravida/ or nullipara/ or pregnant woman/ or primigravida/ or primipara/ or named groups by pregnancy/ or multigravida/ or multipara/ or nulligravida/ or nullipara/ or pregnant woman/ or pregnancy/ or first trimester pregnancy/ or exp multiple pregnancy/ or second trimester pregnancy/ or third trimester pregnancy/ or unplanned pregnancy/ or pregnancy disorder/ or pregnancy complication/ or puerperium/ or weaning/ or lactation/ or pregnancy rate/ or "menopause and climacterium"/ or climacterium/ or early menopause/ or menopause/ or postmenopause/ or premenopause/ | 920094 | Pregnancy, lactation, menopause terms |
| 41 | ("post Tuberculosis sequela*" or "post-tuberculosis sequela*").ti,ab,kf. | 21 |  |
| 42 | or/6-41 | 5666670 | vulnerable population terms |
| 43 | 1 and 5 and 42 | 15622 | Base clinical set 1 - TB and definition and vulnerable populations |
| 44 | limit 43 to (conference abstract or conference paper or "conference review") | 2166 | Conference abstracts |
| 45 | 43 not 44 [***Conference abstracts removed****] | 13456 | Conference abstracts removed |
| 46 | limit 45 to (english or French or Chinese) | 12015 | Language limit |
| 47 | limit 46 to yr="2010 -Current" | 7627 | Publication year limit – Final results |
| 48 | (afghanistan or albania or algeria or american samoa or angola or "antigua and barbuda" or antigua or barbuda or argentina or armenia or armenian or aruba or azerbaijan or bahrain or bangladesh or barbados or republic of belarus or belarus or byelarus or belorussia or byelorussian or belize or british honduras or benin or dahomey or bhutan or bolivia or "bosnia and herzegovina" or bosnia or herzegovina or botswana or bechuanaland or brazil or brasil or bulgaria or burkina faso or burkina fasso or upper volta or burundi or urundi or cabo verde or cape verde or cambodia or kampuchea or khmer republic or cameroon or cameron or cameroun or central african republic or ubangi shari or chad or chile or china or colombia or comoros or comoro islands or iles comores or mayotte or democratic republic of the congo or democratic republic congo or congo or zaire or costa rica or "cote d’ivoire" or "cote d’ ivoire" or cote divoire or cote d ivoire or ivory coast or croatia or cuba or cyprus or czech republic or czechoslovakia or djibouti or french somaliland or dominica or dominican republic or ecuador or egypt or united arab republic or el salvador or equatorial guinea or spanish guinea or eritrea or estonia or eswatini or swaziland or ethiopia or fiji or gabon or gabonese republic or gambia or "georgia (republic)" or georgian or ghana or gold coast or gibraltar or greece or grenada or guam or guatemala or guinea or guinea bissau or guyana or british guiana or haiti or hispaniola or honduras or hungary or india or indonesia or timor or iran or iraq or isle of man or jamaica or jordan or kazakhstan or kazakh or kenya or "democratic people’s republic of korea" or republic of korea or north korea or south korea or korea or kosovo or kyrgyzstan or kirghizia or kirgizstan or kyrgyz republic or kirghiz or laos or lao pdr or "lao people's democratic republic" or latvia or lebanon or lebanese republic or lesotho or basutoland or liberia or libya or libyan arab jamahiriya or lithuania or macau or macao or republic of north macedonia or macedonia or madagascar or malagasy republic or malawi or nyasaland or malaysia or malay federation or malaya federation or maldives or indian ocean islands or indian ocean or mali or malta or micronesia or federated states of micronesia or kiribati or marshall islands or nauru or northern mariana islands or palau or tuvalu or mauritania or mauritius or mexico or moldova or moldovian or mongolia or montenegro or morocco or ifni or mozambique or portuguese east africa or myanmar or burma or namibia or nepal or netherlands antilles or nicaragua or niger or nigeria or oman or muscat or pakistan or panama or papua new guinea or new guinea or paraguay or peru or philippines or philipines or phillipines or phillippines or poland or "polish people's republic" or portugal or portuguese republic or puerto rico or romania or russia or russian federation or ussr or soviet union or union of soviet socialist republics or rwanda or ruanda or samoa or pacific islands or polynesia or samoan islands or navigator island or navigator islands or "sao tome and principe" or saudi arabia or senegal or serbia or seychelles or sierra leone or slovakia or slovak republic or slovenia or melanesia or solomon island or solomon islands or norfolk island or norfolk islands or somalia or south africa or south sudan or sri lanka or ceylon or "saint kitts and nevis" or "st. kitts and nevis" or saint lucia or "st. lucia" or "saint vincent and the grenadines" or saint vincent or "st. vincent" or grenadines or sudan or suriname or surinam or dutch guiana or netherlands guiana or syria or syrian arab republic or tajikistan or tadjikistan or tadzhikistan or tadzhik or tanzania or tanganyika or thailand or siam or timor leste or east timor or togo or togolese republic or tonga or "trinidad and tobago" or trinidad or tobago or tunisia or turkey or turkmenistan or turkmen or uganda or ukraine or uruguay or uzbekistan or uzbek or vanuatu or new hebrides or venezuela or vietnam or viet nam or middle east or west bank or gaza or palestine or yemen or yugoslavia or zambia or zimbabwe or northern rhodesia or global south or africa south of the sahara or sub-saharan africa or subsaharan africa or africa, central or central africa or africa, northern or north africa or northern africa or magreb or maghrib or sahara or africa, southern or southern africa or africa, eastern or east africa or eastern africa or africa, western or west africa or western africa or west indies or indian ocean islands or caribbean or central america or latin america or "south and central america" or south america or asia, central or central asia or asia, northern or north asia or northern asia or asia, southeastern or southeastern asia or south eastern asia or southeast asia or south east asia or asia, western or western asia or europe, eastern or east europe or eastern europe or developing country or developing countries or developing nation? or developing population? or developing world or less developed countr* or less developed nation? or less developed population? or less developed world or lesser developed countr* or lesser developed nation? or lesser developed population? or lesser developed world or under developed countr* or under developed nation? or under developed population? or under developed world or underdeveloped countr* or underdeveloped nation? or underdeveloped population? or underdeveloped world or middle income countr* or middle income nation? or middle income population? or low income countr* or low income nation? or low income population? or lower income countr* or lower income nation? or lower income population? or underserved countr* or underserved nation? or underserved population? or underserved world or under served countr* or under served nation? or under served population? or under served world or deprived countr* or deprived nation? or deprived population? or deprived world or poor countr* or poor nation? or poor population? or poor world or poorer countr* or poorer nation? or poorer population? or poorer world or developing econom* or less developed econom* or lesser developed econom* or under developed econom* or underdeveloped econom* or middle income econom* or low income econom* or lower income econom* or low gdp or low gnp or low gross domestic or low gross national or lower gdp or lower gnp or lower gross domestic or lower gross national or lmic or lmics or third world or lami countr* or transitional countr* or emerging economies or emerging nation?).ti,ab,sh,kf. [****Cochrane - LMIC search terms****] | 2303611 | Cochrane - LMIC search terms |
| 49 | 47 and 48 | 4322 | Cochrane – LMIC results |
| 50 | 47 not 49 [****nonLMIC specific results****] | 3199 | Advanced |

Cochrane (included all languages)

| ID | Search | Hits | Comment |
| --- | --- | --- | --- |
| #1 | [mh ^tuberculosis[mj]] or [mh ^"latent tuberculosis"[mj]] or [mh ^"tuberculosis, pleural"[mj]] or [mh ^"tuberculosis, pulmonary"[mj]] or [mh ^"Mycobacterium tuberculosis"[mj]] | 38 | Tuberculosis terms |
| #2 | (vulnerable or vulnerability):ti,ab | 7163 |  |
| #3 | (definition* or define* or criteria* or "key population" or "high priority" or "high risk" or "at risk" or "disproportionate*" or framework* or dynamic* or susceptibl* or factor or factors):ti,ab | 603629 |  |
| #4 | [mh ^risk] or [mh ^"risk factors"] or [mh ^morbidity] or [mh ^incidence] or [mh ^prevalence] | 39485 |  |
| #5 | {or #2-#4} | 618552 | Definition terms |
| #6 | #1 and #5 ; date range Jan1/2010 to October 29, 2021 | 30 | Final results |

o
